# AI-Assisted Double-Headed Capsule Endoscopy: Multicentre Prospective Diagnostic Accuracy Study Across Small Bowel Indications

**DOI:** 10.3390/diagnostics16020239

**Published:** 2026-01-12

**Authors:** Kamran Mushtaq, Yun Jeong Lim, Cristiano Spada, Alessandro Mussetto, Anastasios Koulaouzidis, Thake Kaung, Dean-Martin Borrow, Cesare Casadei, Praful Patel, Imdadur Rahman

**Affiliations:** 1 Southampton Interventional Endoscopy Unit, University Hospital Southampton NHS Foundation Trust, Southampton SO16 6YD, UK; 2 Department of Internal Medicine, Dongguk University Ilsan Hospital, Dongguk University College of Medicine, Goyang 10326, Republic of Korea; drlimyj@gmail.com; 3 Department of Medicine, Gastroenterology and Endoscopy, Fondazione Policlinico Gemelli I.R.C.C.S., 00168 Rome, Italy; 4 Gastroenterology Unit, Santa Maria delle Croci Hospital, 48121 Ravenna, Italy; 5 Department of Clinical Research, University of Southern Denmark (SDU), 5230 Odense, Denmark; anastasios.koulaouzidis@rsyd.dk; 6 Department of Gastroenterology, Pomeranian Medical University, 70-111 Szczecin, Poland; 7 Department of Gastroenterology, St. Guy’s and Thomas NHS Foundation Trust, Westminster Bridge Road, London SE1 7EH, UK

**Keywords:** capsule endoscopy, artificial intelligence, intestine, small, Crohn’s disease, anemia, iron-deficiency, endoscopy, diagnostic techniques

## Abstract

**Background/Aims:** Double-headed capsule endoscopy enhances visualization and diagnostic yield in small bowel evaluation but increases reading time. This study aimed to assess the diagnostic performance of AI-assisted double-headed capsule endoscopy (MiroCam MC2000) across all small bowel indications and to compare its reading efficiency with the standard manual reading mode. **Methods**: From May to December 2023, 242 consecutive patients (mean age 50.17 years, SD 18.3; 53% female) underwent small bowel capsule endoscopy at two UK centres for suspected Crohn’s disease (48.8%), iron-deficiency anemia (23.6%), bleeding (18.6%), or other (9%). Seven experienced readers reviewed videos in standard mode (blinded to clinical data), then AI-assisted (MiroCam AI Scan) methods were applied after de-identification/randomization. Two experts provided reference standards. No adverse events occurred. **Results:** AI-assisted reading had sensitivity 95.3% (95% CI 90.1–98.3%) and specificity 96.5% (95% CI 91.3–99.0%) for diagnostic findings, vs. standard reading: 96.5% (95% CI 91.2–99.0%) and 85.3% (95% CI 78.0–90.9%). The positive findings rate was 83.6% vs. 80.2% (*p* = 0.040). Reading time decreased by 52% (38.1 vs. 18.26 min; *p* < 0.001). **Conclusions:** AI-assisted reading offers high diagnostic accuracy, superior specificity and reduced reading times, supporting its adjunctive role with expert oversight. Registered: ERGO ID 82419.

## 1. Introduction

Small bowel capsule endoscopy (SBCE) is a key diagnostic tool for small bowel (SB) diseases, particularly for suspected SB bleeding and early Crohn’s disease [1,2,3]. However, the diagnostic yield of SBCE depends on the indications as well as the timing of the capsule, especially for suspected gastrointestinal (GI) bleeding [4,5]. The main challenge with capsule endoscopy (CE) includes the risk of missed lesions and the significant amount of time required to read the study [6]. The average time required to read a study improves with time and expertise, but other factors, such as long SB transit time, reading speed, and mode, can affect the reading time [7,8]. The evidence also suggests that the reader’s efficiency and diagnostic accuracy can sharply decline by just reviewing one reading [9]. The miss rates of CE are variable depending on the number and type of lesions. The current miss rate for different indications, such as polypoidal lesions, is considerably high, up to 19% in some studies [10].

Double-headed video capsules increase diagnostic accuracy, with a clinically significant impact in up to 14.7% of patients [11]. Kim et al. recently showed that the double-tip MiroCam MC2000, providing 340-degree views, has superior diagnostic accuracy compared to the PillCam SB3 for detecting SB lesions, especially clinically significant ones [12]. However, the median reading time was higher than that of PillCam SB3, 30 min vs. 39 min (*p* < 0.001). The European Society of Gastrointestinal Endoscopy (ESGE) has supported the need to develop artificial intelligence (AI)-assisted reading to improve reading time, as well as improve diagnostic accuracy [13]. The use of convolutional neural networks (CNNs) and deep learning models, such as YOLO, is effective in detecting several SB pathologies [14,15]. However, there are a very limited number of real-world studies exploring its use in all SB indications. The American Society for Gastrointestinal Endoscopy (ASGE) AI Task Force has also recently supported the use of AI-based algorithms that can augment the endoscopist’s performance in the detection and characterization of endoscopic lesions [16].

Limited real-world studies explore AI-assisted double-headed capsule endoscopy across all indications. Time savings could reduce costs in high-volume centres, aligning with ASGE and ESGE priorities. Recent artificial intelligence studies have demonstrated promising results, but with important limitations. Xie et al. conducted a large Chinese multicenter study of 2600 single-tip capsule videos showing 95.90% diagnostic accuracy, but this was limited to a single healthcare system and did not evaluate double-tip technology [17]. Spada et al. performed the only other prospective study (*n* = 138) focusing specifically on small bowel bleeding, demonstrating superior artificial intelligence performance compared to conventional reading, but with limited generalizability across all indications [18]. Importantly, both studies used single-tip capsules and were restricted to specific clinical scenarios, leaving a significant gap in understanding real-world artificial intelligence performance across the full spectrum of capsule endoscopy indications using a double-tip video capsule.

We hypothesized that AI-assisted double-headed capsule endoscopy would increase diagnostic yield by ≥10% and reduce reading time by ≥50% compared to standard reading, providing real-world evidence across all indications.

## 2. Materials and Methods

### 2.1. Study Design, Setting, and Ethics

This prospective observational study, adhering to STARD 2015 guidelines, included patients undergoing SBCE at two UK centres (May–December 2023). No industry funding was received; IntroMedic provided devices/software without influencing study design, data, or reporting. Ethical approval was obtained from the University Hospital Southampton IRB (ERGO ID 82419), and all participants provided informed consent.

### 2.2. Patient Enrolment and Exclusion Criteria

Consecutive patients were enrolled to minimize selection bias. Exclusion criteria included inadequate bowel preparation (<25% mucosal visualization) or failure to enter the SB.

### 2.3. Capsule and Preparation Details

The study used de-identified data from SB CE procedures performed with the MC2000 double-headed capsule endoscope. Bowel preparation consisted of a standardized regimen including a clear liquid diet for 24 h before the procedure, followed by overnight fasting. A four-stage visualization score was recorded for each study segment (excellent > 90%, good 75–90%, fair 50–75%, poor < 50% mucosal visualization).

### 2.4. Lesion Definitions and Pre-Study Calibration

We used the International Delphi consensus definitions for vascular, inflammatory, and mucosal abnormalities [19]. Vascular malformations included angioectasia/angiodysplasia, phlebectasia, red spot/red dot, and erythematous patch. Mucosal inflammatory changes comprised aphthoid erosion, deep ulceration, superficial ulceration, stenosis, edema, hyperaemia, and denudation. Normal variants (e.g., lymphangiectasia, cholesterol cysts, nodular lymphoid hyperplasia) were also noted. Before data collection, all capsule readers met for a consensus session with representative images to harmonize interpretation.

### 2.5. Reading Protocol—Standard Mode

**Figure 1:**
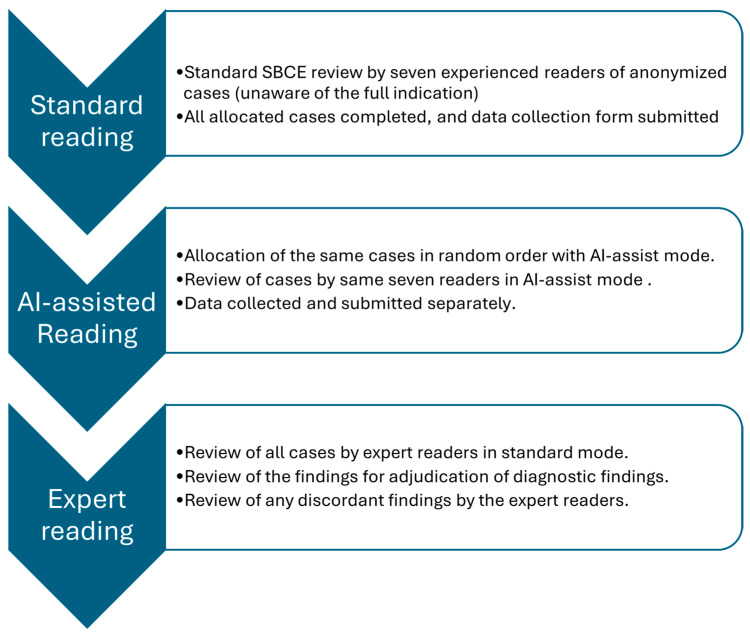
Study methodology flow diagram showing standard and AI-assisted readings by expert and experienced readers.

Experienced readers (with experience of at least 500 capsules) set anatomical landmarks (pylorus to ileocecal valve), then reviewed the study in double-tip camera mode, choosing dual or quad view at up to 20 fps. They recorded only the active review time (excluding annotation and landmarking). Initial standard reads were blinded to clinical context to minimize bias. After stopping the timer, readers labelled and annotated images before completing the data collection tool [20], Figure 1. The cases were distributed among the seven experienced readers through case allocation based on reader availability and workload balancing, rather than all readers reviewing all cases.

### 2.6. AI-Assisted Reading

Following standard mode review, the same videos were reread in random order using MiroCam AI-assist software. Readers again used double-tip mode (at a maximum of 10 fps), recorded the active review time, and completed the data collection tool. The reading speed was limited to 10 to reduce the risk of missing lesions with AI-assist mode due to a significant reduction in redundant images. Readers were allowed to vary the speed but were prevented from exceeding 10 fps. The average washout time period between the standard reading and AI-assisted reading was variable, from a few weeks to months, as the readers read the cases over a couple of months after completion of standard reading. The random order helped to mitigate the recall bias as well.

### 2.7. Expert Reference Reading

Two experts (>2000 SBCE readings each), aware of the full clinical context, read all cases. Their findings served as the reference standard for comparing standard and AI-assisted modes.

### 2.8. Diagnostic and Positive Finding Definitions

Diagnostic rate was the percentage of patients with at least one finding that explained the indication. For overt bleeding, diagnostic findings included bleeding or polypoidal lesions; for IBD, findings suggestive of Crohn’s; for abnormal imaging, corresponding lesions. Positive findings encompassed any lesion of interest, even if they were not diagnostic (e.g., angioectasia in suspected IBD).

### 2.9. Procedural and Demographic Data

Demographics (age, gender, indication), procedural metrics (completion rate, gastric and small bowel transit times, capsule retention), and 4-stage visualization scores were recorded using the data collection tool.

### 2.10. Sample Size Calculation

Studies have demonstrated a 9–14% difference in diagnostic rates between standard and AI modes [17,18]. Using McNemar’s test for paired proportions (difference = 10%, discordant = 0.20, power = 0.9, alpha = 0.05, correlation = 0.3 per literature [16]), 200 patients were required, and this number was inflated to 223 for 10% dropout and subgroup analyses.

### 2.11. Statistical Analysis

Data was analyzed with STATA 18.5 BE Software package. Paired proportions were compared using McNemar’s test. Sensitivity, specificity, and Receiver Operating Characteristic (ROC) curves were calculated against expert readings. Reading times were compared with paired t-tests; Bonferroni correction (alpha = 0.01) adjusted for multiple comparisons. Inter-rater and intra-rater agreement were measured using Cohen’s kappa. A two-sided *p* < 0.05 indicated significance. Standard deviations (SDs) were calculated where applicable.

The inter-rater agreement was assessed using the kappaetc command in STATA, which calculated multiple measures of reliability for categorical data rated by more than two observers. For this study, readers independently evaluated 242 subjects using a binary classification system. Each coefficient was reported with its standard error and 95% confidence interval, providing a comprehensive assessment of both raw and chance-corrected agreement among raters.

Data anonymization procedures included the removal of all patient identifiers, and videos were assigned random study numbers. Cross-border data sharing complied with the UK General Data Protection Regulation (UK GDPR). All international readers completed institutional data sharing agreements and received GDPR training before participation.

### 2.12. MiroCam AI Scan

MiroCam’s abnormal lesion detection AI was developed using a YOLOv4 deep learning architecture specifically designed for object detection, following the methodological framework outlined in “YOLOv4: Optimal Speed and Accuracy of Object Detection” [20]. Training was conducted on a comprehensive dataset of 162,160 images derived from 10,386 clinical CE cases collected from patients from 10 countries at Dongguk University Ilsan Hospital, with data anonymization ensuring patient privacy protection under IRB approvals NODUIH-2018-10-009 (Supplement File S1).

### 2.13. Primary and Secondary Endpoints

The primary endpoint was the Diagnostic accuracy (sensitivity and specificity) of AI-assisted reading compared to standard reading, using expert reading as the reference standard. The secondary endpoint was the difference in the reading time comparison between the two modes. Other endpoints: (a) Diagnostic rate by clinical indication, (b) miss rates, and (c) inter-rater agreement between readers.

## 3. Results

**Figure 2:**
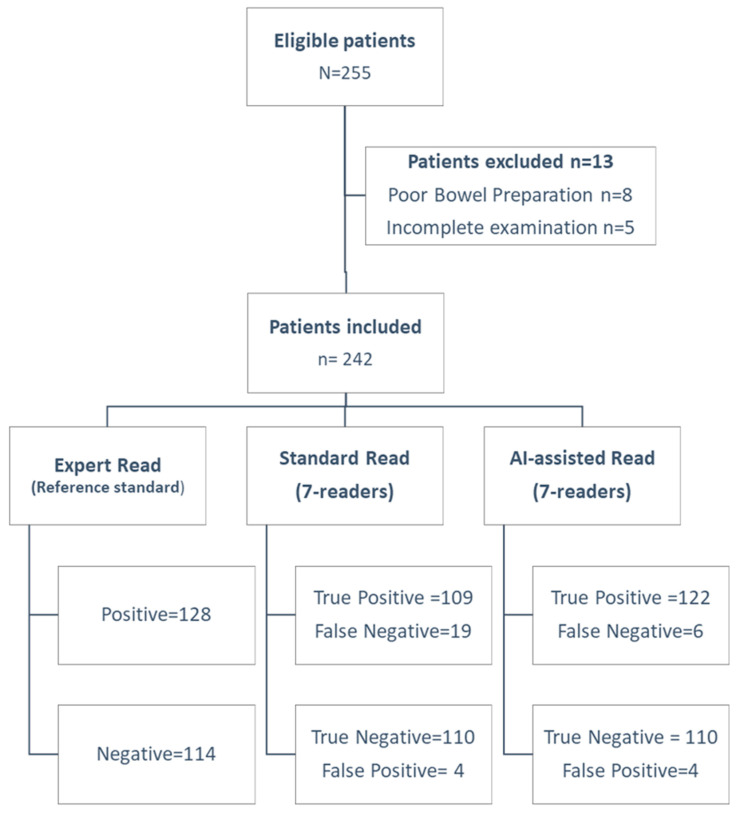
STARD flow diagram of study.

Of 255 patients, 242 were included (mean age 50.17 years, SD 18.3; range 19–91). See Figure 2 (STARD flow diagram) for exclusions. There were more females in our study, 53% females (*n* = 128). The most common indication for CE was suspected Crohn’s disease 48.8% (*n* = 118), followed by unexplained iron-deficiency anemia 23.6% (*n* = 57), suspected SB bleeding 18.6% (*n* = 45), and other factors 9% (*n* = 22), as shown in Table 1. The mean SB transit time for the study was 4.52 h with an SD of 2.15 h (range 0.86–11.5 h). The overall diagnostic rate for experts was 52.9% (95% CI 46.53––59.2%). Standard reading’s diagnostic rate was 46.7% (95% CI 40.3–53.2%; *p* = 0.0026) vs. expert, while the AI-assisted rate was 52.1% (95% CI 45.7–58.5%; *p* = 0.75). AI had a higher positive findings rate, 83.6% (95% CI 78.4–87.8%) vs. 80.2% (95% CI 74.7–84.8%); *p* = 0.04. No capsule retentions or adverse events occurred. The AI-assist mode was statistically better in overall diagnostic rate when compared to standard reading mode, as shown in Table 2.

**Figure 3:**
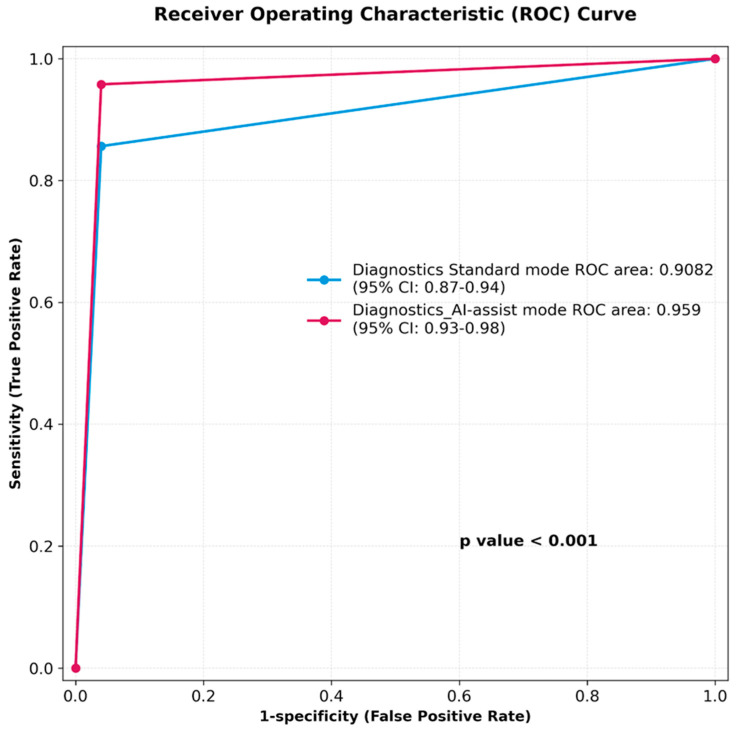
Receiver Operating Characteristic (ROC) curve for standard reading vs. artificial intelligence-assisted reading.

The sensitivity and specificity of MiroCam AI-assisted reading for diagnostic findings were 95.3% (CI 90.1–98.3%) and 96.5% (91.3–99.0%), respectively. The sensitivity and specificity of the standard reading mode for diagnostic findings were 96.5% (CI 91.2–99.0%) and 85.3% (78.0–90.9%), respectively. The MiroCam AI scan (IntroMedic Co Ltd., Seoul, South Korea) had a higher rate of positive findings per patient when compared to standard reading mode, 83.6% vs. 80.2% (*p* < 0.04). Figure 3 summarizes the area under the ROC curve (AUC), showing the superior diagnostic performance of the AI-assisted reading: 0.959 over standard reading mode 0.908 (*p* < 0.001). There were diagnostic findings in 128 patients, and we analyzed the miss rate compared to the expert’s diagnostic rate. Miss rates were 14.8% (95% CI 9.4–22.1%) for standard and 4.7% (95% CI 2.1–9.3%) for AI-assisted reading (Table 3).

**Figure 4:**
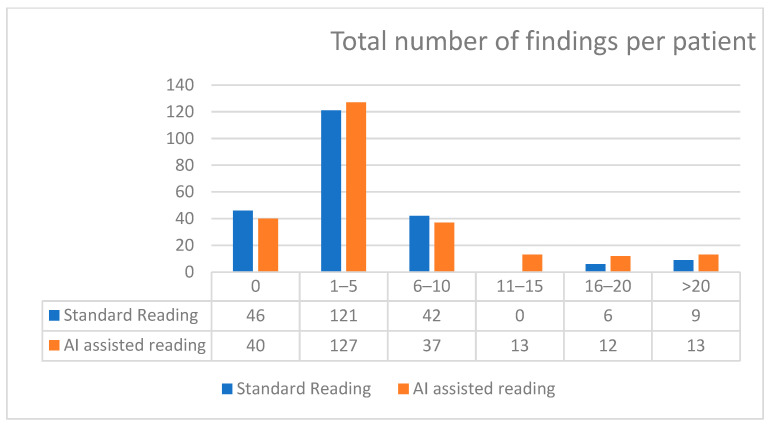
Distribution of total findings per subject (standard vs. AI-assisted reading).

**Figure 5:**
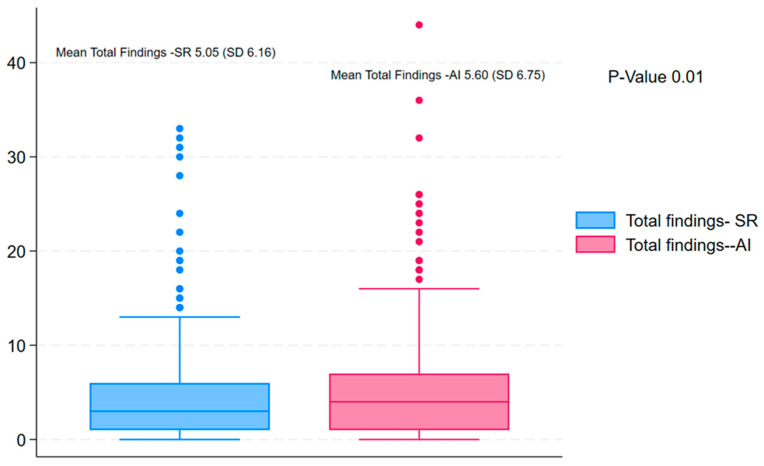
Box plot for the total findings in standard reading mode (blue) compared to AI-assist mode.

The distribution of total findings per subject was compared between standard reading and AI-assisted reading using the Wilcoxon signed-rank test. The results showed a statistically significant difference between the two methods (Wilcoxon signed-rank statistic = 0.0, *p* < 0.001), indicating that the number of findings identified per subject differed between standard and AI-assisted readings (Figure 4). The AI-assisted reading consistently detected a higher number of abnormalities as compared to the standard readings (Figure 5).

Subgroup analysis by indication demonstrated variations in diagnostic rate and positive findings across clinical categories, as seen in Table 4. For suspected IBD, the diagnostic rate for AI-assisted reading was 54.2%, which is marginally higher than standard reading (45.8%) and closely aligned with the expert reading rate (53.4%). This group also exhibited the highest overall positive findings rate at 87.3%. In patients evaluated for unexplained iron-deficiency anemia, AI-assisted and expert readers detected diagnostic findings in 50.9% of cases, compared to 47.4% for standard reading, with a positive findings rate of 79.6%. Suspected small bowel bleeding had notable improvement with AI-assisted reading (55.6%), compared to standard (53.3%) and expert (62.2%) readings, and a positive findings rate of 82.2%. These findings highlight the consistent improvement in diagnostic rates and lesion detection using AI assistance across all major indications for capsule endoscopy. The sensitivity and specificity for the key indications were assessed. There was a significant difference in the sensitivity of AI-assisted reading over standard reading for suspected IBD patients, 98.4% (95% CI 91.5–99.7) vs. 82.5% (95% CI 71.4–90.0), *p*-value 0.0063. The sample size for other indications was underpowered to detect a meaningful difference (Table 5).

**Figure 6:**
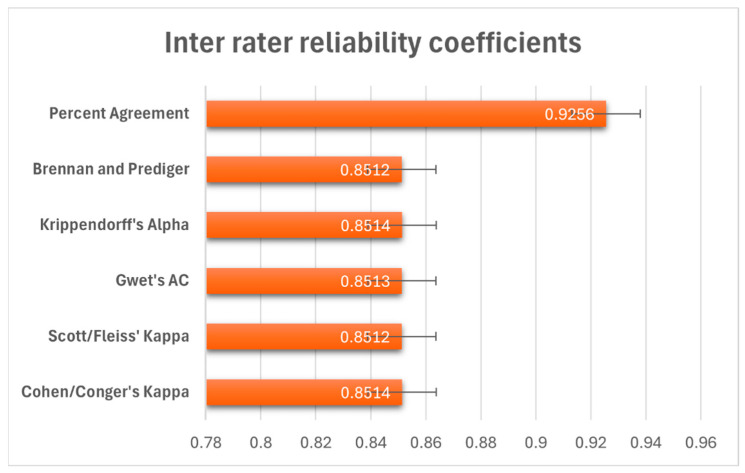
Forest plot of inter-rater reliability coefficients (point estimates and 95% confidence intervals) for percent agreement and chance-corrected statistics. The vertical dashed line at 0.8 indicates the threshold for strong agreement.

We tested how well our readers agreed with each other in terms of the diagnostic findings by having multiple readers assess 242 subjects using a yes/no classification system. The results showed excellent agreement between all readers. Overall, readers agreed 92.6% of the time, as shown in Figure 6. We also calculated several statistical measures that account for agreement happening by chance alone, and these all were within the range of 0.85 (0.80–0.95), indicating strong reliability (Cohen’s kappa = 0.85, along with similar values for other reliability measures (*p* < 0.001).

**Figure 7:**
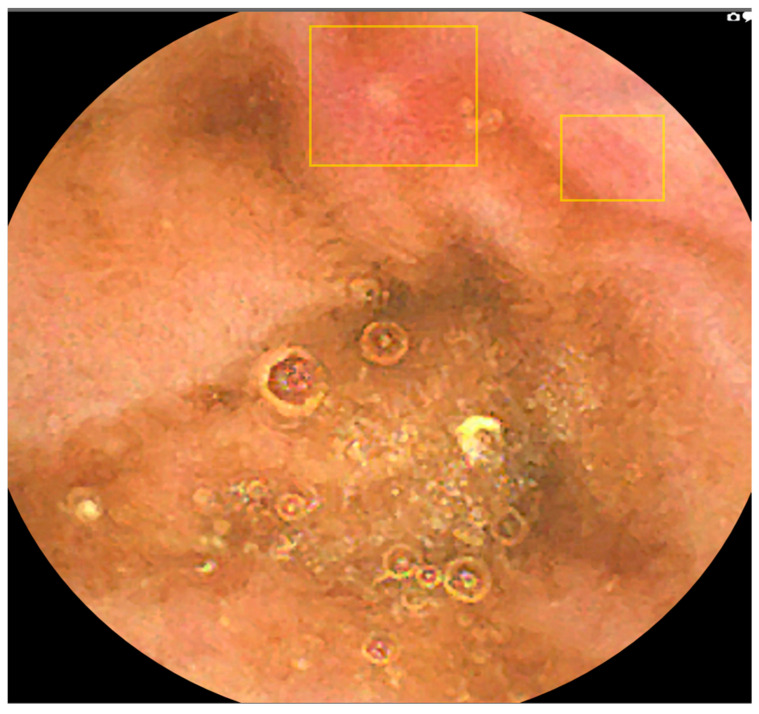
Image example where AI detected a lesion that was not marked during standard reading.

**Figure 8:**
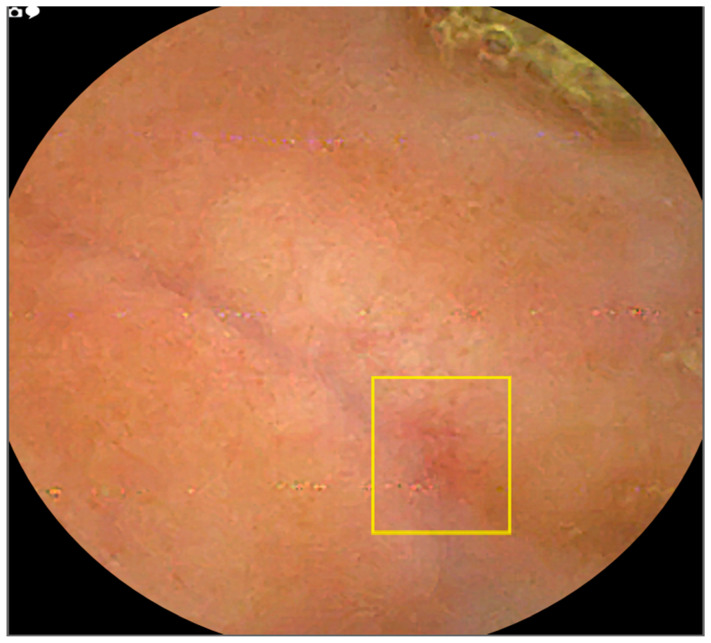
Image example where AI detected an erythematous patch that was not marked during standard reading.

AI-assisted reading mode demonstrated a significant reduction of 6.3 times in image review workload compared to standard reading. The mean number of small bowel images requiring review was 96,700.01 (SD = 46,683.61) for standard reading versus 15,363.91 (SD = 9219.71) for AI-assisted reading. This resulted in a statistically significant difference of 75,336.1 images (77.9% reduction; t = 20.3936, *p* < 0.0001). Figure 7 and Figure 8 illustrate examples of missed lesions identified by AI-assisted scans.

## 4. Discussion

This prospective multicentre study demonstrates that AI-assisted double-headed capsule endoscopy (MiroCam MC2000, Intromedic Co., Ltd., Seoul, South Korea) achieves high diagnostic accuracy, with superior specificity compared to standard reading, across all indications. The diagnostic accuracy of MiroCam’s AI-assisted reading was significantly higher when compared to standard reading. The diagnostic accuracy rate for standard reading when compared to an expert for clinically significant findings, which are deemed diagnostic, was significantly lower. Our results are consistent with the diagnostic accuracy of artificial intelligence-assisted readings by other studies [15,17,18,21].

Recent studies have reported high sensitivity and specificity of AI-assisted scans when compared to standard reading. Xie et al. reported a diagnostic accuracy of the SmartScan-assisted reading of small bowel capsule studies to be 95.90%. This was a large, multicentre, Chinese study with a single-tip capsule review of over 2600 videos [17]. The only other prospective study was by Spada et al., which included 138 patients and showed that the AI-assisted reading was superior (*p* < 0.0001) to conventional reading for detecting SB bleeding P1 and P2 lesions in per-patient analysis. However, their AI-assisted reading was not significantly different when compared to the experts, *p*< 0.060 [18].

The enhanced diagnostic accuracy of AI-assisted reading, particularly for Crohn’s disease (sensitivity 98.4%), may reduce diagnostic delays and expedite treatment initiation, potentially improving patient outcomes such as reduced hospitalization rates or disease progression. It can also facilitate the treat-to-target strategy in patients with already established Crohn’s disease, improving clinical outcomes, as supported by a recent clinical trial [22]. Future studies should evaluate clinical outcomes, such as time to treatment or quality of life, to confirm these benefits.

Our study showed high positive rates of both standard and AI-assisted scans (80.3–83.9%). The difference between the two was still statistically significant, which is also consistent with the findings of other researchers. Xie et al. found a detection rate for their SmartScan-assisted reading compared to standard reading (79.3% vs. 70.7%) [17]. The higher overall positive rate in our study was partly due to the study protocol, which included the detection of minor findings, such as red dots. These were part of the international Delphi consensus on vascular lesions. This might have caused a slightly higher overall positive rate. However, the focus of this study was on clinically relevant diagnostic findings.

The sensitivity of AI-assisted reading was comparable to standard reading overall (95.3% vs. 96.5%), but specificity improved substantially (96.5% vs. 85.3%). In suspected IBD, our largest subgroup, AI, achieved significantly higher sensitivity (98.4% vs. 82.5%; *p* = 0.0063) with identical specificity, while smaller indications lacked power to demonstrate sensitivity differences. Notably, the AI-assisted read was performed after a washout period, with cases presented in random order, minimizing fatigue and recall bias. The marked specificity gain, therefore, likely reflects standardization of lesion interpretation rather than fatigue mitigation.

The progressive development of accurate CNN-based detection of lesions is also important in accurately identifying small bowel lesions [23,24]. The YOLOv4 model’s visualization of suspected lesions via coloured bounding boxes enhances clinical interpretability by directing endoscopists’ attention to regions of interest, as detailed in Supplementary File S1. This feature mitigates ethical concerns about AI’s lack of explainability by providing objective localization, fostering trust in clinical decision-making. For example, in cases of suspected Crohn’s disease, bounding boxes highlighted subtle inflammatory lesions missed by standard reading (Figure 6 and Figure 7).

Studies show that physicians miss about 11% of lesions, and this statistic is greater for mass lesions, at nearly 19% [25]. Per-patient miss rates in our study were 14.8% (95% CI 9.4–22.1%) for standard reading and 4.7% (95% CI 2.1–9.3%) for AI-assisted, though per-lesion rates are unknown, limiting clinical interpretation. This is comparable to other studies, which have shown low rates of miss by AI-assisted scans. In the study by Spada et al., the evaluation of an AI-assisted scan for suspected small bowel bleeding in a cohort of over 138 patients, the miss rate was varied in per-patient and per-lesion analyses, with expectantly higher miss rates with per-lesion analysis (35.2% with standard vs. 12.1% AI-assisted scan) compared to per-patient analysis (21.0% vs. 6.6% for AI-assisted scan). They were specifically looking for higher-risk bleeding lesions. Similarly, the Xie et al. study had a 1.0% missed rate per patient with their SmartScan-assisted reading. The miss rate for their AI scan per patient was 4.1%.

The reading time in our study was significantly reduced to over half of the conventional reading time. The 52% reduction in reading time with AI-assisted reading (18.26 vs. 38.1 min) could yield significant cost savings in high-volume centres, aligning with ASGE and ESGE priorities for efficient resource utilization [13,16]. For instance, reduced reading times may decrease endoscopist workload, allowing higher throughput of capsule endoscopy procedures.

The interobserver agreement in identifying the correct diagnostic findings in our study showed a strong correlation (Kappa 0.85). This is much better than reported in the capsule studies in general [26]. Other AI studies have not reported their interobserver agreement. Several factors have been identified to affect the poor interobserver agreement in reported studies. In their study, Jang et al. had shown that training on the terminology for reporting can significantly improve the interobserver agreement [27]. We believe the consensus meeting and agreement, as predefined in the group, and a clear data collection form, made the reporting of findings very easy for the CE readers, which may have contributed to the poor interobserver agreement [19].

Our study uniquely advances this field by combining double-tip capsule technology with artificial intelligence across all clinical indications, representing a significant methodological advance over prior single-indication or single-tip studies. Unlike previous research limited to specific healthcare systems or bleeding-focused protocols, our real-world prospective multicentre approach demonstrates artificial intelligence effectiveness across the full spectrum of capsule endoscopy applications.

A limitation of this study is the use of expert readers (>2000 capsule reads) as the reference standard, rather than device-assisted enteroscopy, which is considered more definitive for confirming small bowel pathology. However, the current literature suggests comparable diagnostic accuracy between video capsule endoscopy and enteroscopy for key indications like bleeding [22]. Future studies incorporating enteroscopy correlation would strengthen accuracy estimates.

Per-lesion analysis was not performed due to the study’s focus on per-patient diagnostic yield, which aligns with clinical decision-making for capsule endoscopy indications. This limits insights into AI performance for specific lesion types (e.g., angioectasia vs. ulcers). Future studies should include per-lesion analyses to quantify AI’s detection capabilities across pathology subtypes, as demonstrated by Spada et al. for bleeding lesions [18].

This study was conducted in high-volume UK centres with experienced readers (>500 capsules), which may limit generalizability to lower-volume centres or novice practitioners. AI assistance could offer greater benefits in such settings by mitigating reader fatigue, as noted by Leenhardt et al., who highlighted AI’s role in enhancing lesion detection under high cognitive loads [6]. Future research should evaluate AI performance across diverse reader experience levels.

The same readers evaluated all videos in both the standard and AI-assisted modes to enable paired comparisons of diagnostic yield and reading time. This design introduces a risk of recall bias, whereby readers may remember findings from the initial standard review and thereby influence diagnostic performance during the subsequent AI-assisted reading. Randomization of case order for the AI-assisted sessions and distribution of readings over several months were intended to mitigate, but cannot eliminate, this limitation.

As of now, the AI-assisted reading as a standalone in the real world is still not recommended due to the lack of robust evidence supporting its diagnostic accuracy. Ethical concerns include AI’s lack of explainability, necessitating expert oversight for clinical trust. Based on our findings, artificial intelligence-assisted reading demonstrates sufficient accuracy and efficiency gains to warrant clinical implementation in appropriate settings. Artificial intelligence should complement rather than replace human expertise, with manual review capabilities maintained for review of all cases.

Future research should prioritize clinical outcome studies evaluating the impact of artificial intelligence-assisted reading on diagnostic delays, treatment decisions, and patient prognosis. Additionally, studies examining artificial intelligence performance in diverse populations, patients with complex medical histories, and rare small bowel conditions would enhance understanding of technology limitations and appropriate clinical applications.

## 5. Conclusions

AI-assisted double-headed capsule endoscopy demonstrates high diagnostic accuracy and reduced reading times across indications, supporting its adjunctive role with expert oversight pending validation with device-assisted enteroscopy and clinical outcomes.

## Figures and Tables

**Table 1:** Patient demographics and indications for small bowel capsule endoscopy (*n* = 242). Other indication cases: Suspected eosinophilic enteritis (*n* = 1), suspected small bowel graft versus host disease (*n* = 1), protein-losing enteropathy (*n* = 1), and unexplained abdominal pain with suspected small bowel pathology (*n* = 1).

Indications	Frequency	Percent
Suspected Crohn’s disease	118	48.76%
Suspected small bowel bleeding	45	18.60%
Iron-deficiency anemia	57	23.55%
Abnormal imaging	12	4.96%
Small bowel polyposis surveillance	6	2.48%
Other indications	4	1.65%
Total	242	100.00%

**Table 2:** The diagnostic performance of artificial intelligence-assisted reading and standard reading compared to experts as a reference standard.

	Standard Reading Compared to Experts		AI-Assisted Reading Compared to Experts		
Category	Value	95% Confidence Interval	Value	95% Confidence Interval	*p*-Value
Diagnostic Rate	46.70%	40.3–53.2%	52.06%	46.4–59.3%	0.002
Sensitivity	96.50%	91.2–99.0%	95.30%	90.1–98.3%	0.156
Specificity	85.30%	78.0–90.9%	96.50%	91.3–99.0%	<0.001
ROC area	0.91	0.87–0.94	0.96	0.93–0.98	<0.001
Positive predictive value	85.20%	77.8–90.8%	95.30%	89.1–98.1%	<0.001
Negative predictive value	96.50%	91.3–99.0%	94.80%	89.1–98.1%	0.234
Positive findings	80.2%	74.7–84.8%	83.6%	78.4–87.8%	0.040

**Table 3:** Miss rate per-patient analysis for diagnostic findings.

Reading Mode	Misses	Total Expert	Miss Rate	*p*-Value
Standard reading	19	128	14.8% (95%CI 9.4–22.1)	0.009
AI-Assisted reading	6	128	4.7% (95% CI 2.1–9.3)	0.009

**Table 4:** Subgroup analysis showing the diagnostic rates of different reading modes for each indication. Abbreviations: IBD, Inflammatory Bowel Disease; SB, small bowel.

				Diagnostic Rate N (%)				
Indication	N	Mean Age	Female %	Standard Reading Mode *n* (% 95% CI)	AI-Assisted Reading Mode *n* (% 95% CI)	Experts Reading Mode *n* (% 95% CI)	Positive Findings Rate *n* (% 95% CI)	*p*-Value
Suspected IBD	118	40.0	57.6%	54 (45.8%, 37.0–54.7)	64 (54.2%, 45.3–63.0)	63 (53.4%, 44.4–62.1)	103 (87.3%, 80.1–92.1)	0.194
Iron-deficiency anemia	57	60.1	63.2%	7 (47.4%, 35.0–60.1)	29 (50.9%, 38.3–63.4)	29 (50.9%, 38.3–63.4)	45 (78.9%, 66.7–87.5)	0.697
Suspected SB bleeding	45	61.3	33.3%	24 (53.3%, 39.1–67.1)	25 (55.6%, 41.2–69.1)	28 (62.2%, 47.6–74.9)	37 (82.2%, 68.7–90.7)	1.00
Abnormal imaging	12	61.3	25.0%	4 (33.3%, 13.8–60.9)	4 (33.3%, 13.8–60.9)	4 (33.3%, 13.8–60.9)	11 (91.7%, 64.6–98.5)	1.00
SB polyposis surveillance	6	52.0	50.0%	4 (66.7%, 30.0–90.3)	4 (66.7%, 30.0–90.3)	4 (66.7%, 30.0–90.3)	6 (100.0%, 61.0–100.0)	1.00
Other	4	47.0	75.0%	0 (0.0%, 0.0–49.0)	0 (0.0%, 0.0–49.0)	0 (0.0%, 0.0–49.0)	4 (100.0%, 51.0–100.0)	N/A

**Table 5:** Subgroups analysis for sensitivity and specificity by key indications.

Indications	Diagnostics	Standard Reading	AI-Assist Reading	*p*-Value
Suspected IBD	Sensitivity	**82.5%** (71.4–90.0)	**98.4%** (91.5–99.7)	**0.0063**
	Specificity	96.4% (87.7–99.0)	96.4% (87.7–99.0)	
Iron- deficiency anemia	Sensitivity	86.2% (69.4–94.5)	93.1% (78.0–98.1)	0.625
	Specificity	92.9% (77.4–98.0)	92.9% (77.4–98.0)	
Small bowel bleeding	Sensitivity	85.7% (68.5–94.3)	89.3% (72.8–96.3)	1.00
	Specificity	100.0% (81.6–100.0)	100.0% (81.6–100.0)	

## Data Availability

The data presented in this study are available upon request from the corresponding author due to privacy reasons.

## Supplementary Materials

The following supporting information can be downloaded at https://www.mdpi.com/article/10.3390/diagnostics16020239/s1.

## Abbreviations

The following abbreviations are used in this manuscript:

AI	Artificial Intelligence
AI Scan	Artificial Intelligence Scan (MiroCam analysis software)
AI-assist/AI-assisted	Artificial Intelligence–assisted reading mode
AUC	Area Under the Curve
ASGE	American Society for Gastrointestinal Endoscopy
BE	Basic Edition (software version designation in STATA 19.5 BE)
CE	Capsule Endoscopy
CI	Confidence Interval
CNN	Convolutional Neural Network
ERGO ID	Ethical Review Governance Online Identifier
ESGE	European Society of Gastrointestinal Endoscopy
Fps	Frames per Second
GI	Gastrointestinal
GDPR	General Data Protection Regulation
hrs/hr	Hours
IBD	Inflammatory Bowel Disease
IRB	Institutional Review Board
MC2000	MiroCam double-headed capsule model 2000
min	Minutes
ROC	Receiver Operating Characteristic
SB	Small Bowel
SBCE	Small Bowel Capsule Endoscopy
SD	Standard Deviation
STARD	Standards for Reporting of Diagnostic Accuracy Studies
UK	United Kingdom
YOLO	You Only Look Once (object detection model)
YOLOv4	Fourth version of You Only Look Once deep learning architecture
