# Supplementary material for: AI-Assisted Double-Headed Capsule Endoscopy: Multicentre Prospective Diagnostic Accuracy Study Across Small Bowel Indications
Source: Diagnostics (Basel). 2026 Jan 12;16(2):239. doi: 10.3390/diagnostics16020239 (PMC12840249; doi:10.3390/diagnostics16020239)
Supplement: Supplementary file 1 [file diagnostics-16-00239-s001.zip › diagnostics-4033080-supplementary.pdf]

Supplementary File

-Development and Validation of MiroCam's AI software

Model Development

An abnormal lesion detection Artificial Intelligence (AI) was developed using a YOLOv4 deep learning model, which is based on object detection. The development was guided by the paper "YOLOv4: Optimal Speed and Accuracy of Object Detection."(1)

Model Architecture

The YOLOv4 model incorporates the YOLOv3 architecture in the head and uses CSPDarknet53 (Cross-Stage-Partial connections) as the backbone network. For the neck, the model utilizes SPP (Spatial Pyramid Pooling) and PAN (Path Aggregation Network) structures. Additional modules, such as BoF (Bag of Freebies) and BoS (Bag of Specials), are integrated to enhance both the backbone and the detector.

Model Inference

The inference results from the YOLOv4 object detection model are post-processed into a binary classification output to determine whether an abnormal lesion is present.

If the YOLOv4 inference detects one or more suspicious regions (highlighted by yellow bounding boxes) in the input image, the image is classified as an abnormal lesion image. Otherwise, it is classified as a normal image.

Detailed Inference Workflow (as described in the figure 1)

a. The input consists of four images representing bleeding, inflammatory, vascular, and polypoidal lesions.

b. Each input image is in RGB format with a resolution of 320 × 320 pixels and is fed into the YOLOv4 model.

c. The output of YOLOv4 shows that all four images are classified as abnormal lesion images, consistent with their content.

d. For each image classified as abnormal, the model highlights the suspicious region with a yellow bounding box.

e. These yellow bounding boxes serve as visual indicators, similar to heatmaps, offering objective evidence for the AI's decision by showing which part of the image the model focused on during classification.

Application in Small Bowel Lesion Diagnosis

The YOLOv4-based object detection model was trained using Ground Truth data (reference standard) annotated by physicians. This enables the model to assist clinicians in diagnosing lesions in the small intestine.

The YOLOv4 model performs both classification and localization, identifying abnormal lesions and their locations. However, the primary objective of the algorithm is to deliver a binary classification result—determining whether an input image is normal or abnormal.

When a frame is classified as abnormal, the system provides additional support by visualizing the suspected lesion region, helping physicians with objective localization and improving diagnostic confidence

Figure S1: YOLOv4 AI Model Inference Workflow for Abnormal Lesion Detection

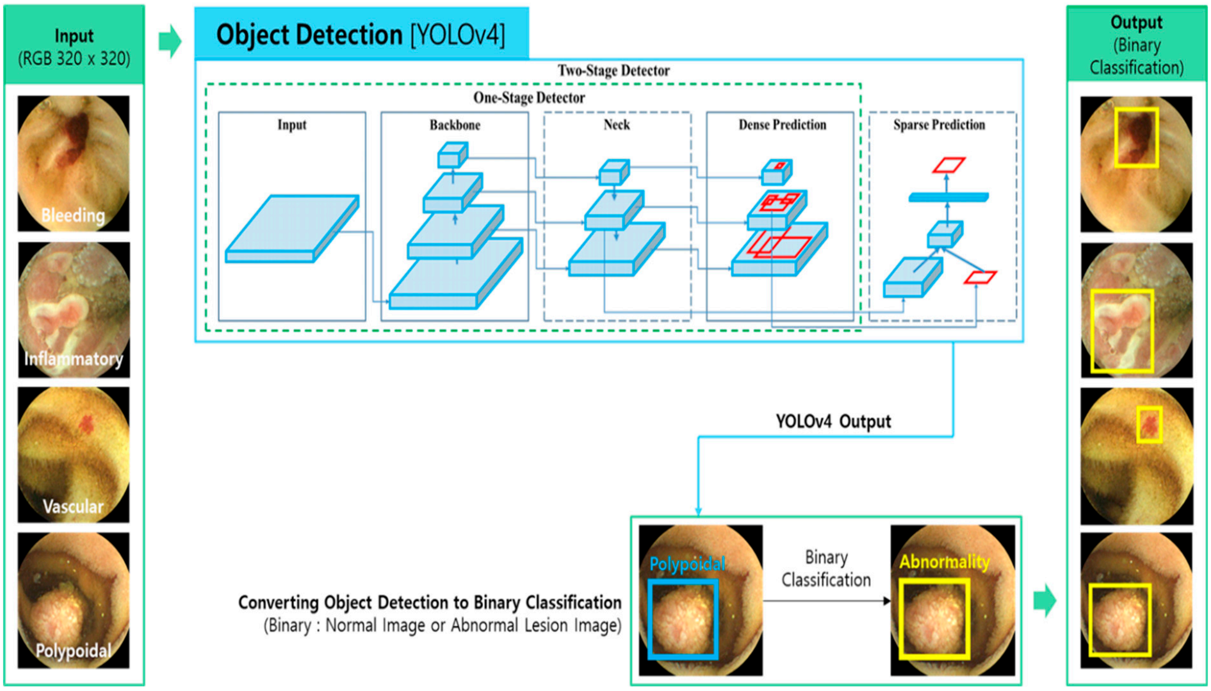

Training Data:

The training was conducted by classifying images into four lesion categories: bleeding, i nflammatory, vascular, and polypoidal. To address the issue of data imbalance among t hese classes, data augmentation techniques were applied to increase the number of im ages in the three minority classes, matching the quantity of the majority class (inflamma tory images).

For external augmentation, various techniques were employed, including image rotation, scaling, translation, cropping, and flipping. Additionally, the YOLOv4 model utilizes Mosaic augmentation, which combines four different images into one during training, further enhancing model generalization and robustness.

Data Source:

A total of 10,386 clinical capsule endoscopy cases were collected from Dongguk University Ilsan Hospital in Gyeonggi-do, South Korea (IRB Registration Number NODUIH-2018-10-009). These cases included both domestic and international patients. Among them, approximately 2,000 cases involved patients with identified lesions, while the remaining cases were classified as normal.

All clinical data were anonymized to ensure patient privacy, with personally identifiable information (such as name, age, and gender) removed before use.

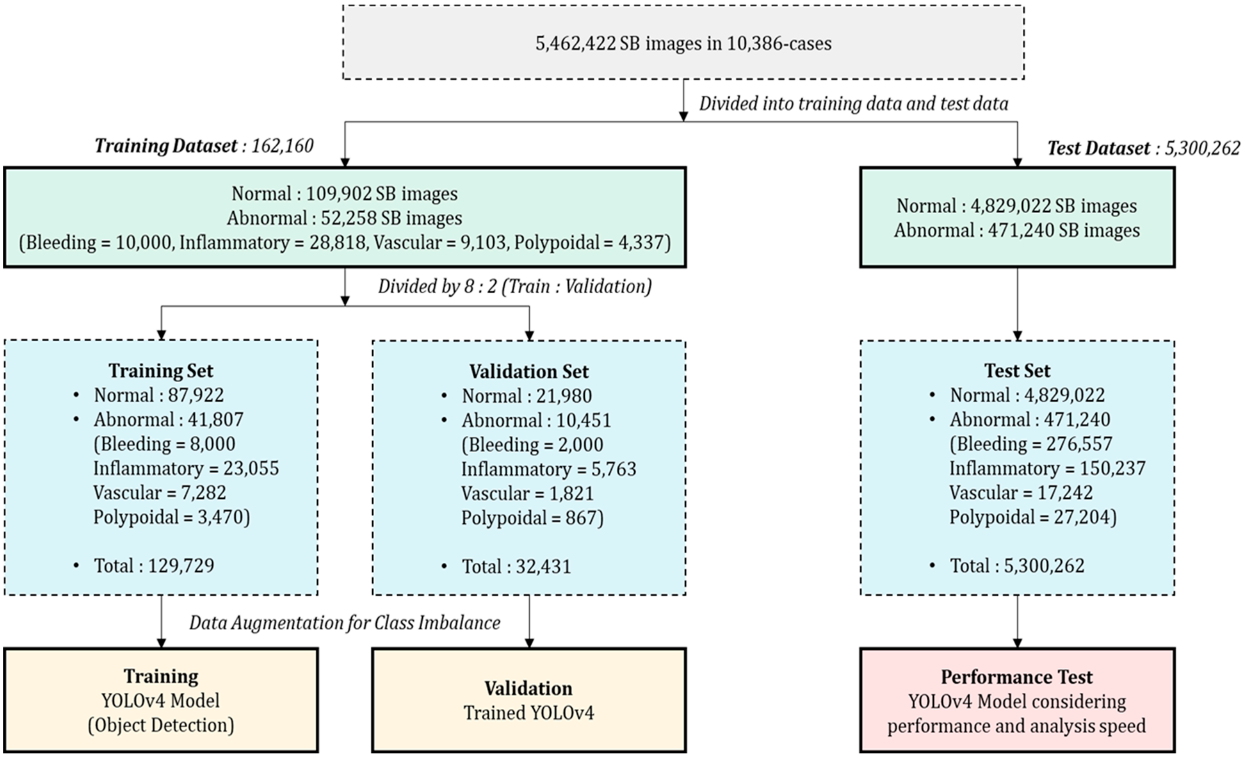

Figure S2: Sample Lesion Categories from Training Data (Bleeding, Inflammatory, Polypoidal, and Vascular Lesions)

Table S1: Data Distribution by Geographic Region and Image Classification (Normal vs. Abnormal Lesions)

Countries	Number of clinical patients	Total number of extracted & labeled images
China	4,311	Normal Images : 4,938,924<br><br>Abnormal Lesion Images : 523,498
UK	1,896	
Portugal	1,314	
Italy	126	
Greece	95	
France	242	
India	82	
Malaysia	1,702	
Korea	618	
Total	10,386	5,462,422

Table S2: Test Dataset Distribution for Binary Classification (Abnormal Lesion vs. Normal)

<table>
<tr><th>Categories</th><th colspan="2">Number of images</th><th rowspan="6"></th><th colspan="2">Classification</th></tr>
<tr><td>Normal</td><td colspan="2">4,829,022</td><td>Normal</td><td>Abnormality</td></tr>
<tr><td>Bleeding</td><td>276,557</td><td rowspan="4">471,240</td><td rowspan="4">4,829,022</td><td rowspan="4">471,240</td></tr>
<tr><td>Inflammatory</td><td>150,237</td></tr>
<tr><td>Vascular</td><td>17,242</td></tr>
<tr><td>Polypoidal</td><td>27,204</td></tr>
</table>

The bleeding, inflammatory, vascular, and polypoidal lesions were grouped into the abnormal lesion image category (4,829,022 images), and normal images were grouped into the normal image category (471,240 images). The binary classification performance of the YOLOv4 model was evaluated based on its inference results for these categories.

Optimal Threshold (TH) Determination

For all lesion categories, the threshold was set to TH = 0.02, the same as in the binary classification performance test.

Figure S3: Data Distribution Across Geographic Regions and Lesion Types

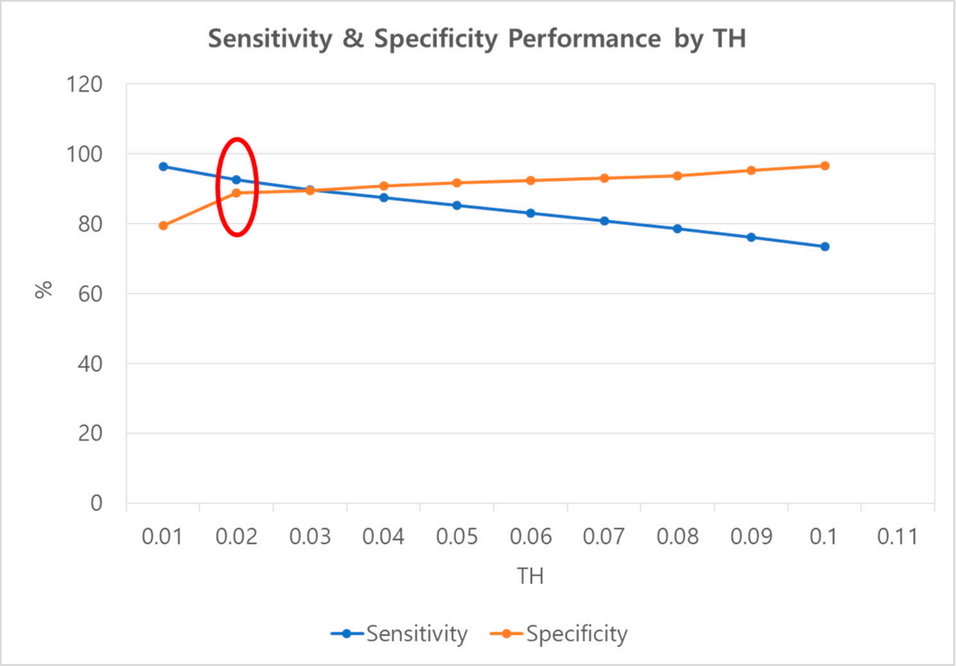

Table S3: Model Performance Evaluation Results by Classification Threshold (TH)

TH	Performance Evaluation		
	Sensitivity (%)	Specificity (%)	Accuracy (%)
0.01	96.45	79.55	80.32
0.02	92.55	88.72	89.01
0.03	89.76	89.37	89.39
0.04	87.41	90.91	90.76
0.05	85.24	91.71	90.63
0.06	83.06	92.31	91.60
0.07	80.82	93.02	91.14
0.08	78.50	93.66	91.46
0.09	76.07	95.26	92.68
0.10	73.53	96.62	94.92

Threshold: For determining whether the area inferred by the YOLOv4 model is classified as an abnormal lesion area

In the example images of the inference results, the blue rectangles indicate the ground truth regions, and the green rectangles indicate the predicted regions.

Figure S4: Model Inference Results with Ground Truth and Predicted Bounding Boxes.

Bleeding

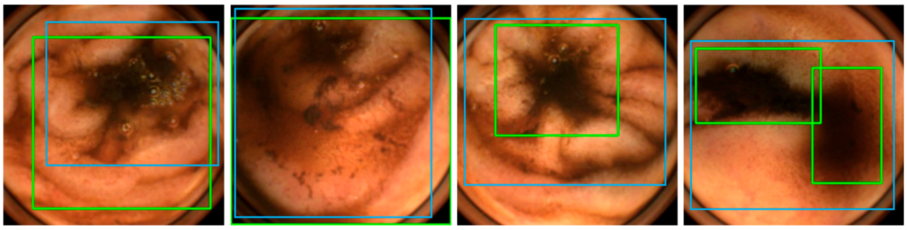

Vascular

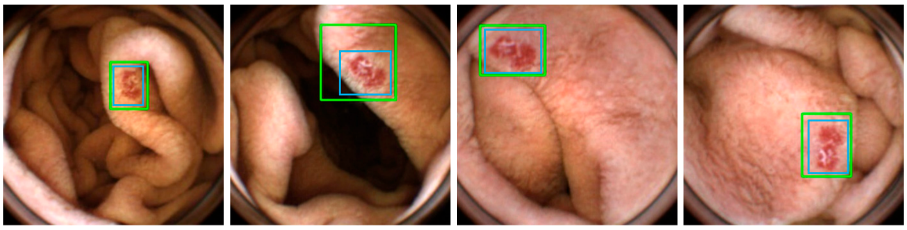

Inflammatory

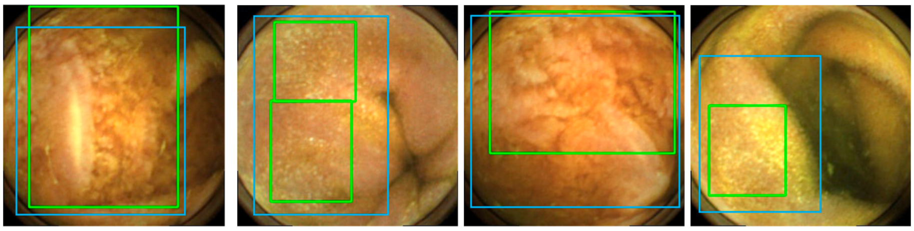

Polypoidal

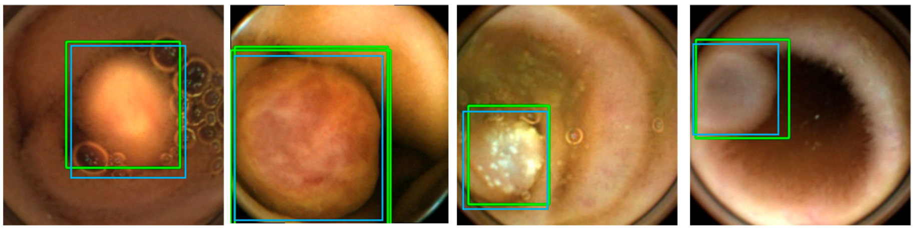

References:

1. Bochkovskiy A, Wang CY, Liao H yuan. YOLOv4: Optimal Speed and Accuracy of Object Detection. 2020.
